# 孤立性肺结节病变的诊断与处理策略

**DOI:** 10.3779/j.issn.1009-3419.2013.09.11

**Published:** 2013-09-20

**Affiliations:** 100021 北京，北京协和医学院中国医学科学院肿瘤医院胸外科 Department of Toracic Surgery, Cancer Hospital, Peking Union Mediacal College & Chinese Academy of Medical Sciences, Beijing 100021, China

**Keywords:** 孤立性肺结节, 诊断, 处理, Solitary pulmonary nodules, Diagnosis, Management

## Abstract

目前，肺癌已跃居成为我国发病率及死亡率最高的恶性肿瘤，总体5年生存率较低；早诊早治是提高肺癌患者生存率及改善预后的关键，而早期肺癌患者常无任何症状和体征，只在影像学上表现为肺孤立性结节病变。提高对孤立性肺结节良恶性的鉴别诊断能力是临床诊治过程中的难点与热点。随着各种诊治技术的发展，孤立性肺结节病变性质的诊断准确率已大大提高。

## Abstract (en)

Currently in China, the incidence and death rate of lung cancer with low five-year survival rates has ranked the first position among all cancers. Early detection of lung cancer and early treatment are of great importance. However, patients with early satge lung cancer always do not present with any synptoms or signs, only to demonstrate solitary pulmonary nodules (SPNs) in radiology findings. So it is very critical to improve the ability to identify the SPNs, and with the development of sorts of diagnostic modalities, the accuracy in the evaluation of the SPNs has imporved greatly.

近年来，随着人们健康意识的提高及影像技术的飞速发展，孤立性肺结节（solitary pulmonary nodules, SPNs）的发现比率正逐年增加；文献报道SPNs的发现率约为8%-51%^[1]^，美国每年估计约新增15万SPNs患者^[2]^。而对于其良恶性质的鉴别及其处理是当今影像科及临床医生研究的热点与难点。目前国际公认的SPNs定义是指肺实质内单发孤立的圆形或类圆形、直径≤3 cm，不伴有肺不张、无淋巴结肿大或肺内其他异常的病变^[2-6]^。

由于定义的把握不同、手术患者选择标准不同及其他相关临床因素各异，SPNs中恶性肿瘤（常为早期肺癌）的概率约为10%-68%^[7]^。目前由于大部分肺癌患者初诊时病变已发展至中晚期，肺癌患者的总体5年生存率仅仅约15.6%^[8]^；而早期肺癌患者术后5年生存率可高达80%^[2, 7]^。因此，提高对SPNs的良恶性鉴别的能力，达到肺癌早诊早治的目标，能极大地提高肺癌患者总体生存率并改善预后。同时，可以避免对良性结节患者采取不必要的有创检查或开胸手术治疗。现将目前国内外对SPNs诊断及处理策略进行综述。

## 1 胸片（chest radiographs, CR or CXR）

目前，很多SPNs患者常常是由普通平片所发现的。病变的大小往往决定了发现的难易程度， < 1 cm的结节常常很难发现^[9]^。国外报道在胸部平片检查中发现肺结节病变的概率约为0.09%-0.2%^[2]^。Dabrowska等^[10]^报道5, 726例常规胸片检查中，检查出SPNs（116例）概率为2.2%，其中良性病变为44%，恶性为21%，病变最小径为4 mm。平片能够提供病变位置、大小、生长速度、边缘特征、有无钙化、空洞、卫星灶等相关信息，而这些特点在某种程度上能够将良恶性病变予以鉴别^[7, 9, 11, 12]^，尤其是在随访期间的胸片间的对比^[10]^。随着CT的广泛普及，加上平片中重叠效应的致命弱点，很多医生往往并不重视平片在发现及鉴别SPNs的作用，从而降低了在胸片中发现SPNs的能力；Huston报道^[13]^在行胸片检查（包括透视和胸片多次对比）中，对SPNs的诊断的准确性可达67%。同时，相关技术的改进也提高了鉴别诊断的准确性^[14-16]^。2011年，Freedman等^[17]^报道使用一种新型软件程序对肋骨、锁骨、胸骨等组织进行抑制而产生修改后的图像，从而使诊断恶性的灵敏度由49.5%提高至66.3%。2012年，Xu等^[18]^报道评价利用实时交互肺结节分析系统的胸部数字X片对肺癌患者进行筛查，其发现SPNs的灵敏度可达77.3%，从而提出此分析系统是一种能可以进行大规模人群肺癌筛查的有效手段。因此，胸部平片仍应作为常规检查，不断提高的数字胸片诊断技术将为未来进行大规模早期肺癌筛查提供帮助。

## 2 计算机断层扫描（computed tomography, CT）

由于CT有着更高的分辨率、敏感性、特异性，加上横断面扫描克服了重叠效应的弱点，其在SPNs鉴别诊断上得到广泛应用，已成为首选的影像鉴别手段。来自一份多中心研究结果表明，当将CT强化值定义为15 HU时，CT的敏感性及特异性分别为98%、58%^[19]^。最大径 > 1 cm、无钙化、密度不均匀、分叶毛刺、位于上叶、病变增大、伴胸膜凹陷及血管集束征等都提示SPNs为恶性肿瘤的可能^[20]^。

### 2.1 形态学特点（morphologic features）

#### 2.1.1 大小

SPNs的直径大小对良恶性质的鉴别有着至关重要的作用。恶性肿瘤的大小与其生长速度密切相关，直径越大，意味着恶性的可能性越高；直径指的是结节长度和宽度的平均值。根据Swensen的一项前瞻性研究^[21]^显示， > 4 mm的结节恶性的概率不到1%；4 mm-7 mm、8 mm-20 mm、20 mm-30 mm恶性的概率分别约为0.9%、18%、50%^[22]^；在结节的恶性似然比（likelihood ratio, LR）中，直径≤1 cm、1.1 cm-2.0 cm、2.1 cm-3.0 cm分别为0.52、0.74、3.67^[23]^。因此，SPNs的直径大小可以作为鉴别良恶性的独立影响因子。

### 2.2 内部特征（internal characteristics）

#### 2.2.1 钙化（calcification）

钙化可被认为是将良性病变从恶性中鉴别出来的最重要的影像特点。文献^[24]^报道在504例钙化结节中良恶性概率分别为97%（490）、3%（14），1, 109例无钙化结节中良恶性概率分别为29%（327）、71%（782）。良性结节钙化形态常有中央、分层、弥漫、爆米花样四种，前三种形态往往是肉芽肿性炎（如真菌感染或结核等）表现；爆米花样钙化常为错构瘤的特点，概率甚至可以达到100%^[4, 25, 26]^。但是仍有约2%-13%的结节型肺癌、33%的类癌等恶性肿瘤存在钙化，常常表现为无定形、点状或偏心状；原因包括肿瘤坏死后相邻肉芽组织包裹、骨肉瘤及软骨肉瘤或粘液分泌型癌转移瘤等^[6, 23, 25]^。同时，由于38%-63%的良性结节、67%的类癌及大部分肺癌并不存在钙化^[4]^，此时则需借助其他影像特征来进行鉴别。

#### 2.2.2 脂肪（fat）

结节中出现脂肪成分往往是良性病变的诊断标准之一。CT值约为-40 HU--120 HU的脂肪可出现在50%的错构瘤中。然而，仍有少部分恶性肿瘤可含有脂肪成分，如脂肪肉瘤及肾癌的肺转移瘤^[27, 28]^，结合患者既往肿瘤病史即可诊断。

#### 2.2.3 空泡或空洞征（cavitation）

空泡指结节内1 mm-3 mm的小灶性透光区，是癌灶内部分肺泡未受累及，癌细胞呈覆壁生长而使该部肺泡仍保持完整充气状态。文献报道^[29]^空泡征在 < 3 cm肺癌中的出现率为28.7%，其中直径≤2 cm肺癌占60.4%；而良性病变出现率仅为8.5%。随着肿瘤增大，残存肺组织逐渐被肿瘤组织取代，空泡继续坏死形成癌性空洞。而空洞征在良恶性结节中均可发生，包括脓肿、感染性肉芽肿、wegener's肉芽肿病、肺梗死乃至7 mm的结节型肺癌^[4]^；鉴别主要在于洞壁的厚度及腔内是否规则，不规则且壁厚 > 16 mm的结节恶性概率为84%-95%，而光滑薄壁结节（< 4 mm）近95%为良性^[6, 23]^。然而，Woodring等^[30]^报道壁厚为5 mm^-1^5 mm的空洞结节中良恶性病变概率分别为51%、49%；故直径为此范围的空洞结节鉴别存在一定困难。

#### 2.2.4 空气支气管征（air bronchogram）

肺癌出现此征象比良性病变更加普遍，有时可表现为泡沫样透亮区或假空洞征改变；Kui等^[31]^报道在132例SPNs中，约30%的肺癌（33/115）出现空气支气管征，而良性病变（1/17）仅为5.9%。空气支气管征的出现往往提示着病变多为细支气管肺泡癌、淋巴瘤、结节病、机化性肺炎、肺梗死等^[4, 6, 25]^。然而，国内一篇报道^[32]^回顾分析190例病理证实的外周型SPNs，发现肺癌及良性病变出现空气支气管征的概率分别为58%、52%，认为不能帮助鉴别良恶性结节。

### 2.3 边缘特征（edage characteristics）

#### 2.3.1 分叶、毛刺（lobulated or spiculated）

良性病变常表现为边界清楚、规则、无明显分叶及毛刺；反之，则提示着恶性病变，原因在于恶性肿瘤细胞在肺间质内不均衡生长，可表现为放射冠状或日冕征^[25]^；Swensen等^[33]^用Logistic回归分析建立临床预测模型后认为，分叶征象可视为恶性结节独立的风险因素；其阳性预测值可达88%-94%^[6]^。Furuya等^[34]^分析193例SPNs的影像特点，出现分叶、不规则、毛刺的结节分别约97%、93%、82%为恶性。同时，Lindell等^[35]^分析5年肺癌筛查经验，随着时间的推移，发生进展的肿瘤42%出现边缘变化，而其中80%的改变为分叶、毛刺增多。然而，仍有近25%的良性结节出现分叶及部分炎性病变出现毛刺改变，如肉芽肿性炎、类脂性肺炎、机化性肺炎、结核、肺纤维化等^[4, 23]^，但毛刺常较长、较细；另外，约21%的恶性结节并没有明显分叶及毛刺，尤其是肺转移性肿瘤^[26]^。

#### 2.3.2 胸膜凹陷征（pleural indentation）

近年来，国外对此研究及报道少于国内；其形成的基础在于病变内间质纤维成分（常为瘤内成纤维母细胞产生纤维瘢痕）牵拉所致，同时由于负压吸引生理性液体积聚于胸膜凹陷处，从而在影像学上表现为喇叭影。一般认为，此征象是恶性结节的特异性表现之一，诊断肺癌阳性率约81%，且其分型在鉴别诊断中有重要作用^[36-38]^；但是国内一项*meta*分析^[39]^显示胸膜凹陷征诊断直径 < 3 cm的周围型小肺癌不具有特异性。与伴有胸膜凹陷的良性结节的鉴别在于胸膜是否增厚、粘连及牵拉影是否均匀。吴华伟等^[40]^认为，影响胸膜凹陷征形成的主要因素依次为瘤内纤维化、瘤/壁距离、肺癌组织类型。

#### 2.3.3 血管集束征（vessel convergence）

血管集束征是指一支或数支血管走行肿瘤边缘或穿过肿瘤所形成的征象，往往为肺癌的征象之一，其病理基础被认为是恶性肿瘤细胞产生血管形成因子，诱发新生血管或原有供血血管增粗以向肿瘤供血。恶性SPNs此征象出现率约为83%-94.8%；由于各学者报道的血管集束征分型不同，从而诊断恶性SPN的特异性也有所不同。有人提出支气管血管集束征概念，并认为血管集束征并非肺癌的供血血管和肿瘤血管，形成气管血管集束征的基本原因是肿瘤瘤体内纤维化灶和肿瘤增殖破坏致使肺结构的塌陷皱缩。虽然部分良性结节也可出现血管集束征，但此征象的出现首先应考虑恶性^[41-43]^。

#### 2.3.4 卫星灶（satellite lesions）

SPNs周围出现卫星灶，常常是肺内肉芽肿性炎的特点；其良性病因的阳性预测值为90%^[6]^。但是，约6%-10%的恶性结节周围可出现卫星灶，由原发病灶肺内转移所致^[23]^，即临床病理分期中的T3。

### 2.4 生长速度（growth rates）

结节的生长速度常用其容积倍增时间来评估，不使用直径主要在于恶性病变增长的不对称性。比较前后CT，若病变容积可见明显增大，恶性可能几乎100%，其倍增时间常在30天-400天左右^[44]^；但若在30天内病变迅速增大，则应考虑感染、炎症或淋巴瘤及部分转移瘤（骨肉瘤或绒毛膜癌肺转移瘤）。倍增时间超过400天常提示良性肿瘤或肺部感染后形成肉芽肿性炎可能^[45]^；过去认为SPNs随诊2年未见其容积增大，则考虑良性病变；但Yankelevitz等^[46]^报道其阳性预测值、敏感性、特异性仅为65%、40%、72%。而且，2年未增长并不适用于 < 2 cm的恶性结节（常为细支气管肺泡癌或高分化的腺癌及典型类癌），因为这些病变出现容积倍增的时间可长达4年^[2, 45]^；Hasegawa等^[47]^报道约20%恶性结节的容积倍增时间超过2年，并且纯磨玻璃样结节（ground-glass opacity, GGO）、混合型结节及实性结节的平均容积倍增时间分别为813天、457天、149天；吸烟者的时间明显短于非吸烟者。Winer等^[48]^对Ⅰ期肺癌的容积倍增时间研究显示，中位倍增时间为181天（25天-1, 212天），22%的肺癌容积倍增时间超过465天，只有6%肺癌1年之内未见明显变化。

### 2.5 位置（location）

SPN的位置是肺癌的一项独立预测因素，恶性结节多位于右肺及上叶，研究显示70%的肺癌发生在上叶，可能与吸入的致癌物容易积聚在上叶有关；而良性病变则可均衡地分布于整个肺内；周围型病变以腺癌居多，而鳞癌常为中心型^[6, 23, 49]^。Lindell等^[35]^利用CT筛查出60例肺癌，位于右肺、左肺、上叶、下叶、右上、左下的比例分别为59%、41%、56%、44%、31%、25%。另外，胸膜下不规则小的良性病变在老年人肺尖特别常见，而毗邻胸膜裂缝下3 mm-9 mm的成角或软圆形小结节常为肺内淋巴结^[50]^。

### 2.6 CT值

CT值指的是X线穿过组织被吸收后的衰减值，以衡量组织对于X光的吸收率。来自一项多中心研究^[51]^显示，以强化衰减值15 HU为临界， < 15 HU时良性病变阴性预测值为96.4%，而 > 15 HU诊断恶性病变的敏感性、特异性、准确性、阳性预测值分别为98%、58%、77%、68%；Yi等^[52]^以30 HU为临界，其敏感性、特异性、准确性、阳性预测值、阴性预测值分别为99%、54%、78%、71%、97%；但两研究的特异性均不高，主要是在于活动性炎症、动静脉畸形或错构瘤强化时CT值衰减较低；为提高特异性，Jeong等^[53]^采用增强剂流入时的 > 25 HU，流出时在5 HU-32 HU之间，诊断恶性特异性可提高到90%。然而，对于直径 < 5 mm结节，或含有脂肪、空洞、坏死、钙化的结节，CT值的应用有效性有待商榷^[45]^。

### 2.7 技术改进

随着CT影像技术的发展，出现了靶扫描、CT灌注成像技术及相关后处理技术如多平面重建（multiplanar reformation, MPR）。从而在多个层面、任意角度充分地显示结节病灶边缘特征及内部征象，更清楚显示结节病变影像解剖结构与毗邻组织之间的关系；而且灌注成像既能提供结节的形态学信息及结节内部血流参数及强化的时间一密度曲线等多种生理学信息，结合SPN形态特点，从而为定性、定位诊断及下一步诊疗方案提供指导意义。另外薄层CT（1 mm）的出现也使SPN（尤其是 < 1 cm的微小结节）诊断的准确性得到提高，研究^[54]^显示，与5 mm CT对比，结节的直径、钙化、密度均匀性两者均有明显统计学差异，1 mm CT能够更加准确地提供结节的鉴别诊断信息。

文献^[55]^报道，综合利用上述特征及相关技术，同时结合患者相关的高危因素如高龄、吸烟史、家族史等，SPN恶性诊断的敏感性及特异性分别可达95.2%、100%。

### 2.8 纯GGO/GGN

之所以专门列出磨玻璃样结节，是因为这种病变常并不具有上述实性结节的特点。其影像学表现为无明显实性成分或中央呈实性而周围磨玻璃样改变，病理基础是肿瘤细胞沿肺泡间隔生长，肺泡壁增厚，但肺泡腔未完全闭塞，内可有少量黏液或脱落的肿瘤细胞；病理常为细支气管肺泡癌、不典型腺瘤样增生、高分化腺癌以及部分非特异性纤维化或机化性肺炎。报道^[56]^显示，75%GGO为细支气管肺泡癌（bronchioloalveolar carcinoma, BAC）或细支气管肺泡癌为主的腺癌，6%为不典型腺瘤样增生（atypical adenomatous hyperplasia, AAH），约19%为良性病变。磨玻璃样改变的比例越少，其恶性可能性则越高，淋巴结转移及术后复发可能性也越大。

## 3 核磁共振成像（magnetic resonance imaging, MRI）

一直以来，MRI在SPN的诊断上应用远不及CT，主要在于其费用的昂贵、低空间分辨率、低信噪比、磁敏感伪影等。但随着近年来MRI软硬件的发展及快速成像系列、磁共振波谱（magnetic resonance spectrum, MRS）、及磁共振扩散加权成像（diffusion weighted imaging, DWI）等的应用，加上无电离辐射性，其在SPNs的诊断应用及敏感性已大大提高，尤其是在SPNs的内部成分、周围毗邻关系和增强后各成分信号特征变化方面有着独特的优势，甚至在SPNs的微血管密度、组织间隙、血管内皮生长因子表达上有重要的鉴别作用^[57]^。

Ohno等^[58]^报道，恶性SPNs的平均相对增强率和平均增强斜率明显高于良性SPNs而低于活动性炎症（*P* < 0.05）；若以最大相对增强率0.15作为鉴别良、恶性结节的阈值（> 0.15提示恶性），0.8作为鉴别恶性SPNs和活动性炎症的阈值（< 0.8提示恶性），则恶性SPNs诊断的灵敏度、特异度、阳性预测值、阴性预测值及准确度分别为100%、75%、88%、100%、91%；若以增强斜率0.025/s作为鉴别良、恶结节的阈值（> 0.025/s提示恶性），0.15/s作为鉴别恶性结节和活动性炎症的阈值（< 0.15/s提示恶性），则恶性SPNs诊断的灵敏度、特异度、阳性预测值、阴性预测值及准确度分别为100%、85%、93%、100%、95%。另外，最大增强比率时间也是一项具有重要鉴别诊断意义的MRI参数。Kono等^[59]^通过回顾性分析202例SPNs的特点，发现85%的恶性SPNs（122/144）最大增强比率时间≤4 min，而所有结核（15例）和错构瘤（12例）的最大增强比率时间均 > 4 min或逐步强化而没有峰值时间（*P* < 0.000, 1）。

因此，MRI在SPNs的良恶性鉴别上有着较大的应用空间。然而，有研究者^[60]^通过对ROC曲线分析，在SPNs的鉴别意义上将其与CT进行对比，认为尽管动态增强MRI在某些方面优于增强CT，但两者在良恶性鉴别诊断方面并没有统计学差异。

## 4 放射性核素显像

### 4.1 正电子发射型计算机断层显像（positron emission computed tomography, PET）

其应用的理论基础是恶性肿瘤的葡萄糖代谢高于正常组织，常采用葡萄糖的类似物18F标记的F-2-脱氧-D-葡萄糖（^18^FDG），被肿瘤组织代谢摄取而达到显像的目的。虽然费用昂贵限制其使用，但此手段已逐渐被公认为目前鉴别SPNs良、恶性的最佳无创手段，尤其是2000年与CT设备整合为PET/CT后，诊断恶性SPNs的灵敏度、特异度分别可达97%、85%，明显高于PET和CT的单独检查手段（*P* < 0.05）^[61]^。大多数研究报道显示常以最大标准摄取值（maximum standardized uptake values, SUVmax）2.5为诊断阈值，SUVmax≥2.5提示恶性。Bryant等^[62]^报道585例直径≤2.5cm SPNs的PET结果，SUVmax值为2.6-4.0、≥4.1其恶性的概率分别为80%、96%。但同时也指出SUVmax值在0-2.5之间仍有24%的恶性可能。SUVmax≤2.5的恶性常为细支气管肺泡癌、类癌及部分高分化腺癌、转移瘤等。因此有学者^[63]^提出采用滞留指数RI（延迟SUVmax/早期SUVmax-1）来进行鉴别诊断，若RI > 10%，SUVmax≤2.5 SPNs良恶性鉴别的灵敏度、特异度、准确性分别为73%、80%、78%, 并提出进一步延长延迟时间（如5 h）则更有助于提高准确性。另外，假阳性可发生于肉芽肿性炎、真菌感染、或结核等活动性炎症。对于直径≤1 cm的SPNs，SUVmax≥2.5诊断恶性的准确性并不高，文献^[64]^报道其灵敏度、特异度、准确性分别为85%、36%、54%。最近，国外已经开始将MR与PET进行设备融合，整合为PET/MR，目前尚未见其在SPNs鉴别诊断方面相关研究结果的报道。

### 4.2 单光子发射计算机断层显像（single-photon emission computed tomography, SPECT）

其基本原理同PET-CT，只是所用放射性标记不同。Nikoletic等^[65]^利用^99m^锝甲氧基异丁基异腈（^99m^Tc-MIBI）SPECT/CT来鉴别SPNs，其敏感度、特异度、准确度、阳性预测值、阴性预测值分别为90%、76.6%、79.4%、88.5%、83.3%；费用低廉、简便易行正促使此项检查在各地逐渐开展。

### 4.3 生长抑素受体显像（somatostatin receptor imaging, SSTR）

在诊断SPNs性质方面是一种相对较新的技术，除各种神经内分泌细胞肿瘤外，多种肿瘤细胞均表达较高的生长抑素受体，利用放射性核素标记的生长抑素类似物（常用的^99m^Tc-depreotid）与受体结合而达到显像的目的。而肺癌组织中的SSTR表达较多，一项多中心实验研究显示，其在诊断SPNs性质灵敏度、特异度分别可达96.6%、71.3%^[66]^。其假阳性主要可见于肉芽肿、错构瘤及间质性肺炎等；另外其吸收比率的不同也可提示SPNs的病理类型，小细胞肺癌的吸收率可高于腺癌及大细胞癌。美国FDA已批准将其用于SPNs的良恶性鉴别诊断，用于筛选需行有创性诊治的SPNs患者。

虽然目前的非有创性检查的诊断灵敏度、特异度各有不同，然而一项*meta*分析研究^[67]^显示，CT、MR、FDG-PET及SPECT等之间在诊断SPNs良恶性质中并没有明显的统计学差异。

## 5 血清学检查

### 5.1 蛋白

目前常用如下几种肿瘤标志物：CEA、CA125、CA199、CA153、Cyfra21-1。文献报道^[68]^恶性SPNs的肿瘤标志物数值明显高于良性SPNs（*P* < 0.05），CEA、CA125、CA199诊断恶性SPNs的灵敏度分别约为55%、36%、41%，特异度分别为80%、95%、85%；而CA153灵敏度可达86%（以24 IU/mL为阈值）。Cyfra21-1被认为是检测肺鳞癌的首选肿瘤标志物，而SPNs病理类型多为腺癌，因而其在诊断SPNs良恶性上并没有较大的应用价值。

### 5.2 血浆分泌型磷脂酶A2-Iia（secretory phospholipase A2-IIa, sPLA2-Iia）

研究显示，肺癌患者血浆中的sPLA2-Ⅱa显著增高。Kupert等^[69]^报道显示将血浆中sPLA2-Ⅱa诊断阈值定为2.4 ng/mL，其诊断T1 SPN的灵敏度及特异度分别为48%、86%，而诊断T2肺癌的灵敏度可升至67%，如联合CEA等其他肿瘤标志物则诊断准确性更高。因此，sPLA2-IIa在未来可能成为新的诊断肺癌的血浆中肿瘤标志物的成员，为SPNs的鉴别诊断提供帮助。

### 5.3 微小RNA（microRNA, miRNA）

近年来，越来越多的研究瞄准于miRNA（血或组织），SPNs良恶性的诊断也不例外。Shen等^[70]^报道，同良性SPNs患者和健康人相比，恶性SPNs患者血浆中miR-21、miR-210明显高表达，而miR-486-5p明显低表达（*P* < 0.001），综合这三种RNA在诊断SPNs病变性质上其灵敏度、特异度分别可达75%、84.95%。然而由于血浆中循环miRNA谱的多样及复杂性，其应用于SPNs的诊断上仍须进一步多中心临床试验验证。

## 6 经皮肺穿刺活检或针吸活检（fine-needle aspiration biopsy, FNAB）

对于不愿通过外科手术诊治明确SPNs病变性质的患者来说，FNAB不失为一种较好的获取病理的方式，尤其是对于位于肺周围离胸壁较近的SPNs。常用三种手段引导：荧光镜（透视）、CT、超声。以定位准确的CT引导下穿刺应用较广，适用于穿刺5 mm以上的SPNs。一项*meta*分析^[71]^报道FNAB诊断恶性SPNs的灵敏度及特异度分别为86%、98.8%，而同时联合透视及CT引导下进行穿刺其灵敏度、特异度可达91%、94%^[72]^。穿刺的准确性与病变所在的位置深度及大小有明显相关性；对于 < 2 cm的SPNs来说，CT引导下穿刺总的诊断准确性约为77.2%^[73]^；而对于直径约0.5 cm-0.7 cm的结节来说，其灵敏度只有50%；同时，针吸的组织样本多少也严重影响病变性质的诊断，对于直径 < 1 cm的结节来说只有约77%病变采样能满足病理诊断^[74]^；Tsukada等^[75]^报道直径6 mm^-1^0 mm、11 mm^-2^0 mm、21 mm-30 mm诊断的准确性分别为66.7%、78.9%、86.7%。FNAB的常见的并发症气胸、出血，其发生的概率分别约为15%、1%^[76]^，其他较少见的并发症包括气体栓塞、肿瘤种植转移等。

## 7 纤维支气管镜（fiber optic bronchoscopy, FOB）

利用FOB对SPNs获取病理诊断的方法包括支气管细胞刷检（BB）、肺泡灌洗（BAL、BW）及经支气管肺活检（TBLB）；文献报道^[77]^在常规FOB检查下，三者的诊断准确性分别仅为41%、40%、52%，而三者联合诊断SPNs良恶性准确度可提高至60%；而当SPN直径≤2 cm时，尤其是位于肺野外1/3时，其诊断准确性则可降至为14%（2/14）。超细支气管镜、支气管内超声及电磁导航引导辅助技术的发展进一步提高了TBLB的灵敏度。

支气管内超声（endobronchial ultrasonography, EBUS）引导下进行活检的原理是在支气管内对结节进行超声定位，利用探针或导向鞘GS对病变进行穿刺获取病理。Herth等^[78]^一项前瞻性研究报道在54例无法进行常规TBLB的SPNs患者中，利用EBUS引导下48例（89%）能予以准确定位，38例（70%）成功进行活检明确诊断。Yamada等^[79]^利用EBUS-GS在对106例SPNs进行活检后分析，直径 < 1.5 cm、1.5 cm-3.0 cm的SPNs诊断准确性分别为40%、76%，同时提出为最大限度提高诊断准确性，应至少进行5次活检采样。

电磁导航支气管镜（electromagnetic navigational bronchoscopy, ENB）是最新发展起来的一项用于诊断肺周围型病变性质的新型技术，其原理是利用磁导航系统，利用CT扫描三维成像制定虚拟导航线路，将支气管镜准确引导至肺周围指定部位以进行活检。已发表的相关研究^[80]^报道ENB诊断SPNs的准确性约为54%-75%；另有学者^[81]^指出ENB下导管针吸诊断恶性SPNs准确率明显高于单纯活检钳（*P* < 0.05），并提出同时结合EBUS其诊断准确性可提高至93%。

## 8 电视辅助胸腔镜手术（video-assisted thoracoscopic surgery, VATS）

对于经上述诊断方法仍不能确诊而患者坚决欲行手术治疗时，VATS可考虑作为新的首选手段，以达到明确诊断及治疗双重目的。目前已逐渐成为SPNs的主要或标准的诊治手段；其诊断的准确性可达100%，并发症发生率、死亡率也较常规开胸手术低^[82, 83]^。良性结节在明确诊断的同时也完成了治疗，消除了患者的心理负担和定期复查的费用；对于恶性SPNs，在术中明确诊断后可直接进行肺癌的根治性手术治疗。同时，文献^[84, 85]^报道，VATS下肺癌根治术后5年生存率与传统开胸手术治疗并无统计学差异，并且创伤小、术后疼痛轻、恢复快、住院时间短。Whitson等^[86]^对早期非小细胞肺癌（non-small cell lung cancer, NSCLC）术后（3, 256例常规开胸手术、3, 114例VATS）生存率进行分析，得出术后4年生存率分别为71.4%、88.4%（*P* < 0.05），总的并发症发生率分别为31.2%、16.4%（*P* < 0.05），同样5年生存率却无统计学差异（*P* > 0.05）。然而，对于目前VATS下诊治SPNs，术中定位存在一定困难，尤其是难以触及的肺内深部病变、 < 1 cm结节、GGO等，目前文献^[87]^报道的技术包括手指探查定位、影像解剖位置定位、CT引导留置Hook Wire定位针、亚甲蓝注射、放射性示踪剂注射、术中超声定位等，均有利弊。

然而，对于早期（Ⅰ期）肺癌的SPN患者的手术方案的仍存有争议。肺叶切除、亚肺叶切除（包括解剖性肺段切除及非解剖性肺楔形切除）在早期肺癌术式选择的地位备受关注。2012年，Stefani等^[88]^回顾性分析了将82例楔形切除及124例肺叶切除的T1N0的NSCLC患者，发现术后并发症、死亡率无明显差异，但两者的局部复发率、5年生存率、5年无疾病生存率存在明显差异，分别是（22%, 8%）、（74%, 85%）、（62%, 77%）。Wolf等^[89]^也认为在能耐受肺叶切除的患者中，肺叶切除仍应作为首选，而亚肺叶切除在年老或肺功能差的患者中值得考虑。同样，一项population-based研究^[90]^支持了这一点，在14, 473例Ⅰ期NSCLC患者中，肺叶切除总生存率及肿瘤相关生存率均明显高于解剖性肺段切除，提出肺叶切除仍应作为Ⅰ期NSCLC标准术式且不考虑肿瘤大小。然而，有学者^[91]^综合分析1988年-2008年（分三个阶段，三阶段手术方式的选择不一）≤2 cm的Ⅰa期NSCLC的手术方式与术后生存率的关系变化，提出肺叶切除是否作为≤2 cm的Ⅰa期NSCLC的标准术式应进行重新评估，他们发现在近些年来的Ⅰa期NSCLC病例中，肺段切除及肺楔形切除后患者的总生存率与肺叶切除相当；2012年，Zhong等^[92]^比较了≤2 cm的Ⅰa期肿瘤患者中行胸腔镜下段切除和叶切除两种手术方式，发现术后并发症、术后局部复发率、5年生存率及无疾病生存率均未见明显差异。而Fan的一篇*meta*分析^[93]^显示，Ⅰ期NSCLC患者行肺叶切除的总生存率和肿瘤相关生存率明显高于亚肺叶切除（*P*=0.000, 6），而 < 2 cm的Ⅰa期患者，总生存率没有差异（*P*=0.58）。而同肺段切除相比，肺叶切除的总生存率（*P*=0.45）和肿瘤相关生存率（*P*=0.97）在Ⅰ期NSCLC中没有明显差异。换言之，亚肺叶切除的术式范围应为解剖性段切除，而不是非解剖性楔形切除。目前，两项比较局部切除和肺叶切除的随机研究正在日本、美国进行，期待能为早期肺癌手术方式选择提供一定的依据。

## 9 处理策略

目前对于SPNs患者如何进行标准化的处理流程并没有完全统一，放射科、呼吸科、胸外科的临床医生处理意见各一^[94]^。

对于≤8 mm的SPNs，Fleischner学会^[95]^推荐对SPNs观察原则是：（1）没有肺癌危险因素（吸烟史；年龄≥60岁；有肺癌史或肺外其他癌病史）的CT随访：①SPN直径 < 4 mm，无须随访，但患者必须完全知情随访的利与弊；②4 mm-6 mm的SPN，隔12个月随访1次，若无变化无需随访；③6 mm-8 mm的SPN，6个、12个、18个、24个月各随访1次，无变化者可停止随访；（2）具有1个以上肺癌危险因素的CT随访：①直径 < 4 mm的SPN，隔12个月随访1次，若无变化无需随访；②4 mm-6 mm的结节，6个、12个、18个、24个月各随访1次，无变化的可停止随访；③6 mm-8 mm的结节，3个、6个、9个、12个月各随访1次，若无变化在24个月再随访1次，无变化可停止随访。

美国胸外科医师协会（ACCP）于2007年在《Chest》杂志上发表了第二版《肺癌诊断和治疗指南》，新指南中重点对SPN的处理策略给予了详细的建议^[96]^；现予以简要总结：

**1**    对于每一个性质不定的SPN患者，至少有胸部CT影像资料，推荐增强薄层CT。

**2**    临床医生根据临床经验或其他信息做出SPN良恶性可能性的评估。

**3**    推荐复习回顾或对照患者以前的胸部和其他的影像学或临床资料。；

**4**    影像学表现明显提示恶性的SPN，若无禁忌症，推荐VATS诊治，VATS诊治较困难时，可行诊断性的外科开胸手术。

**5**    有明显钙化等清晰良性特征的SPN，可3个、6个、12个、24个月CT随访，2年后每年1次。

**6**    SPN稳定在2年以上者，不建议做进一步的诊断性评估，但表现出GGO改变，须每年一次的长期CT随访。

**7**    性质不定的SPN（> 1 cm），可推荐使用PET-CT，若提示恶性，且无禁忌症，建议VATS；若提示良性，可定期随访（每3个月，期间可行抗炎抗结核等治疗），或推荐FNAB、FOB（EBUS、ENB）。

**8**    FNAB、FOB结果显示为恶性或虽FNAB、FOB结果阴性但患者期望明确诊断而不愿再随访时，若无禁忌症，推荐VATS诊治；VATS诊治较困难时，可行诊断性的外科开胸手术。

**9**    FNAB、FOB结果提示恶性而患者拒绝手术治疗或存在明显禁忌时，可行放化疗。

**10**    VATS术中楔形切除SPN，冰冻提示良性则行楔形切除；若提示恶性且能耐受，则行肺叶切除及系统性纵膈淋巴结清扫。

**11**    术中诊断为小细胞肺癌的SPN患者，若无淋巴结转的证据且能耐受手术，推荐手术切除及系统性纵膈淋巴结清扫，行术后辅助化疗。

目前，临床上的SPNs患者越来越多，诊断方法日新月异，但如何更好结合临床上实际情况来选择相应诊治手段、第一时间明确SPNs良恶性质、更好的进行个体化诊治及形成科学合理的诊治规范流程等是未来值得探讨的重要课题。
